# Determinants of Low Fifth Minute Apgar Score among Newborn Delivered in Jimma University Medical Center, Southwest Ethiopia

**DOI:** 10.1155/2020/9896127

**Published:** 2020-03-04

**Authors:** Bekalu Getachew, Tesema Etefa, Adissu Asefa, Behailu Terefe, Diriba Dereje

**Affiliations:** ^1^Department of Biomedical Sciences (Anatomy), Institute of Health Sciences, Jimma University, Jimma, Ethiopia; ^2^Department of Biomedical Sciences (Anatomy), Collage of Health and Medical Sciences, Debre Berhan University, Debre Berhan, Ethiopia; ^3^Department of Pharmacy (Clinical Pharmacy), Institute of Health Sciences, Jimma University, Jimma, Ethiopia; ^4^Department of Biomedical Sciences (Physiology), Institute of Health Sciences, Jimma University, Jimma, Ethiopia

## Abstract

**Background:**

Apgar score is currently an accepted method for newborn infant assessment immediately after delivery. Low fifth minute Apgar score was strongly associated with the risk of neonatal and infant death. Even though much has been done, still, the levels of neonatal mortality in sub-Saharan African countries including Ethiopia were significant. Therefore, this study is aimed at identifying the risk factors so as providing strategies for decreasing the morbidity and mortality of newborns and identifying determinants of low fifth minute Apgar score among newborns delivered in Jimma University Medical Center, Southwest Ethiopia, 2018.

**Method:**

Institution-based cross-sectional study was conducted involving 366 neonates delivered at Jimma University Medical Center. Data was collected by using interview questionnaire. Apgar score was assessed by standard tool at the 1st, 5th, and 10th minutes after birth and only 5^th^ minute Apgar score was used as outcome variable. Consecutive sampling technique was used to select the participants. The collected data were analyzed using SPSS version 20.0. Chi-square test was done at bivariate level and *P* value was used to select candidate variables for multivariate analysis. Finally, a 95% confidence interval was used to assess significance.

**Results:**

A response rate of this study was 95%. The proportion of low 5th minute Apgar score in this study was 11.5%. Prolonged duration of labor (AOR = 15.18, 95% CI: 5.51-40.27), maternal history of khat use (AOR = 3.21, 95% CI: 1.26-8.85), and low birth weight (AOR = 1.65, 95% CI: 1.02-3.11) were predictors of low fifth minute Apgar score.

**Conclusion:**

About one tenth of newborns were having low 5th minute Apgar score. The likelihood of low 5^th^ minute Apgar score was found to increase with prolonged duration of labor, history of mother's khat use, and low birth weight.

## 1. Introduction

The Apgar score was proposed in 1952 as a means of rapidly evaluating the clinical status of newborn infants [1] and currently remains an accepted method for newborn infant assessment immediately after delivery [2]. Reduced Apgar score, including less than five Apgar score components, is as valuable as the full Apgar score to predict neonatal mortality [3].

Low Apgar scores was associated with increased perinatal morbidity and mortality [4]; neonatal and infant death had a strong association with low Apgar score (<3) [5]. In infants with low Apgar score at 5 min, there is increased risk of neonatal respiratory distress, need for mechanical ventilation and admission to neonatal intensive care unit [6], and also, a higher risk of childhood cancers [7].

Children with a five-minute Apgar score of 3 or less and signs consistent with neonatal encephalopathy had a significantly increased risk of developing minor motor impairments [8]. Low cognitive function, neurologic disability, and even subtle cognitive impairment as measured by academic performance at 16 years of age was showed to be associated with Apgar scores of less than 7 at five minutes after birth as evidenced in recent studies [9, 10]. According to the study done in the USA, low Apgar score was associated with increased mortality in premature neonates, including those at 24–28 gestational weeks of age, and proposed to be a useful tool for clinicians in assessing prognosis of newborn [11].

The heart rate and respiratory effort components at 5 min are central for neonatal mortality [3]. Infants with low 5-minute Apgar score have greater risk for mortality and morbidities associated with prematurity [12]. Of infants with a very low Apgar score at 5 minutes, 81% had a poor outcome. In these infants, the relative risk (RR) for perinatal mortality was 24.93 (13.16-47.20) [13]. Deaths within 24 hours were more common among infants with Apgar scores 0–3 than among infants with Apgar scores 7–10 [14]. The risk of developmental vulnerability at 5 years of age is inversely associated with the 5 min Apgar score across its entire range and even the score can serve as a population-level indicator of developmental risk [15].

According to sustainable development goals (SDGs), under five mortality, is planned to be reduced by 50% in WHO African region by specifically reducing newborn mortality by relying on better prevention and management of preterm birth, inpatient care, and support of newborn and postneonatal period [16]. During the Millennium Development Goal (MDG) era, many countries in Africa achieved marked reductions in under-5 and neonatal mortality. Yet, the pace of progress toward these goals substantially varied at the national level, demonstrating an essential need for tracking even more local trends in child mortality [17]. In Ethiopia, although the target for under-five mortality rate was achieved, the targets for infant and neonatal mortality rates were just below the MDG four targets [18]. Despite a 50% reduction in childhood mortality, reduction of early neonatal death (ENND) has significantly lagged behind other Millennium Developmental Goal achievements and is a growing contributor to overall mortality in children aged <5 years.

Therefore, deaths in the newborn period and especially ENND are increasingly contributing to overall infant mortality at age <5 years [19]. Even though much has been done, still, the levels of neonatal mortality in sub-Saharan African countries were significant. One of the early neonatal assessment tools for neonatal status is Apgar score. Therefore, identifying determinants of low Apgar score is very important in early neonatal prevention of morbidity and mortality. There were no adequate published researches in Ethiopia that identified predictors of fifth minute Apgar score. Thus, this study was aimed at assessing determinants of low fifth minute Apgar score among newborn delivered at Jimma Medical Center. In addition, this study will give a new dimension in identifying the risk factors, and also provides an input for the prevention and early identification of those risks for decreasing the morbidity and mortality of newborns.

## 2. Methods

### 2.1. Study Area and Period

This study was conducted in Jimma University Medical Center (JUMC) which is located in Jimma town, Jimma zone, 355 Km in the southwest of Addis Ababa, the capital city of the Ethiopia. JUMC is a 500-bedded teaching and referral hospital that provide general and specialized clinical services including maternal and childcare for about 15 million populations in southwestern part of Ethiopia. The Gynecology/Obstetric wards of the hospital, with 45 maternity beds and five delivery tables, provide 24-hour obstetrics and gynecology care to both direct and referral cases. The study was conducted from May 1 to May 30, 2018.

### 2.2. Study Design

An institution-based cross-sectional study was conducted among neonates delivered at JUMC.

### 2.3. Source Population

All neonates delivered at JUMC.

### 2.4. Study Population

All neonates delivered at JUMC and presented during data collection period.

### 2.5. Eligibility Criteria

#### 2.5.1. Inclusion Criteria

- All mother/neonate index singleton live births after 28 weeks of gestation during the study period were included in the study

#### 2.5.2. Exclusion Criteria

- Those neonates with mothers who were seriously ill
- Those neonates whose mothers were not willing to give information
- Deliveries of unknown gestational age

### 2.6. Sample Size and Sampling Technique

A sample size of 366 was calculated using single population proportion formula using a 35.7% proportion of 5th minute low Apgar score from previous study in Ethiopia [20] at a 95% confidence limit, 5% margin of error, and correction formula. Consecutive sampling technique was used.

(1) $$\begin{array}{c} n=\frac{{\left(z_{\alpha /2}\right)}^{2}\cdot pq}{d^{2}}, \end{array}$$

where *n* is the sample size, Z^?^/_2_ is the confidence interval = 1.96, *P* is prevalence = 35.7%, and *d* is margin of error = 5%.

With the above assumptions, the calculated sample size is 352.

Since the estimated number of deliveries was less than 10,000, we use correction formula.

(2) $$\begin{array}{c} \text{nf}=n/\left(1+\frac{n}{N}\right). \end{array}$$

nf is the final sample size for the targeted population, *n* is the calculated sample size (352), and *N* is the total targeted population (6000).

Then, the final sample size according to these equation yields 333, and adding 10% for nonresponse (33), it becomes 366.

### 2.7. Study Variables

#### 2.7.1. Operational Definition

- Fifth minute Apgar score: Apgar score of newborn neonate at fifth minutes immediately after delivery

### 2.8. Data Collection Tool and Procedure

The interview questionnaire was adapted after review of different literatures. It consisted of socio-demographic, maternal behavioral factors, maternal obstetric variables, and neonatal characteristics. Completeness of the data and relative accuracy of Apgar score estimation was evaluated by a senior midwife.

Data was collected by two midwifery nurses in the delivery and operation rooms. Training on the standard procedures of Apgar score was provided to data collectors. Each newborn recruited was assessed for Apgar in the 1st, 5th, and 10th minutes after birth and weighed only once soon after delivery.

Apgar score was estimated using five variables, strength and regularity of heart rate (100 beats/minute or more (2 points), less than 100 (1 point), none (0 points)), lung maturity/breathing effort (regular breathing (2 points), irregular and <30 breath/minute (1 point), none (0 points)), muscle tone and movement (active (2 points), moderate (1 point), limp (0 points)), skin color/oxygenation (pink (2 points), bluish extremities (1 point), totally blue (0 points)), and reflex response to irritable stimuli (crying (2 points), whimpering (1 point), silence (0 points)). Then, the score of each finding was summed up by an investigator and a score below value seven was considered as low. The data was checked on a daily basis by data collectors and further overseen by the principal investigator for completeness and correctness during collection periods. It was also rechecked during data entry. Training was given for three days on the purpose of data collection, techniques of data collection, and on ways of bias minimization. The questionnaires were prepared in English and translated in to Amharic and retranslated back to English to check its consistency.

### 2.9. Data Analysis

The collected data were checked for its completeness and then entered to Epi data version 3.1 (Atlanta, US) then exported to SPSS version 20.0 for analysis. Frequencies, means, standard deviation, and percentage were used for the descriptive analysis of data. Chi-square test was done at bivariate level and *P* value was used to select candidate variables for multivariate analysis. Finally, a 95% confidence interval was used to assess significance.

### 2.10. Ethical Consideration

Ethical clearance was obtained from the Institutional Review Board (IRB) of Jimma University. Supportive letter was written to JUMC. The purpose of the study was explained to the neonate mothers briefly. Data collection was started after permissions were obtained from hospital managers/medical directors and after a written consent was obtained from children's mothers. Data that were collected from the participants were kept in secured and locked cabinets in order to maintain confidentiality. Voluntary participation was ensured and participants are free to opt out.

## 3. Results

### 3.1. Sociodemographic Characteristics

A total of 348 neonate/mother pairs were involved in this study yielding a response rate of 95%. About 75.6% of the mothers were aged 21–34 years. Majority 62.4% of the participants were married. Nearly one third (29.6%) of the mothers were illiterate. About 38.8% of the mothers were housewives. Almost half of 50.9% the participants were Oromo by ethnicity. About 52.3% of the participants were from rural area. Majority of 46.6% participants earn monthly income of 1000-2800 ETB (Table 1).

### 3.2. Maternal Obstetric-Related Characteristics

Majority (71.6%) of the deliveries were spontaneous. Normal duration of labor was reported in 85% of the cases. Most (93.7%) of the mothers had history of spontaneous abortion. Regarding mode of delivery, about 42.8% of deliveries were spontaneous vaginal delivery. About 5.5% and 7.8% of the mothers had history of stillbirth and infant death, respectively. ANC follow-up was reported in 91.1% of the mothers. Almost half (50.6%) of the mothers attended ANC 2-3 times. One-fifth of the mothers had history of complication during current pregnancy. About 27% of the mothers had history of chronic illness during pregnancy (see Table 2).

### 3.3. General Neonatal Characteristics

More than half (51.1%) of neonates were females and about 44.3% of them were third order and above. About 64.7% of neonates were term deliveries. Regarding their presentation, 65% of them were vertex. Most of (81.3%) the neonates had normal birth weight. The proportion of low fifth minute Apgar score was 11.5% (see Table 3).

### 3.4. Behavioral and Nutritional Characteristics

About 13.5% of the mothers had passive smoking history, 16.1% had Khat chewing history, and 17% had alcohol use history. Folic acid use was reported by 7.5% of the mothers (see Table 4).

### 3.5. Factors Associated with Low Fifth Minute Apgar Score

From bivariate analysis, eleven variables were candidates (*P* value < 0.25) for multivariate regression analysis: maternal education (*P* value, 0.113), gravidity (*P* value, 0.129): duration of labor (*P* value, 0.211), history of stillbirth (*P* value, 0.179), number of ANC visits (*P* value, 0.113), sex of neonate (*P* value, 0.028), smoking (*P* value, 0.172), maternal khat use (*P* value, <0.00), fetal presentation at birth (*P* value, 0.014), condition of labor (*P* value, 0.196), and birth weight (*P* value, 0.011) (see Table 5).

### 3.6. Independent Predictors of Low Fifth Minute Apgar Score

Neonates born from mothers who had a normal duration of labor were 24% likely to have low Apgar score (AOR 0.24; 95% CI, 0.22-0.55) than those with prolonged duration. Mothers who had history of khat use were 3 times more likely to give birth to babies with low Apgar score than those without khat use (AOR = 3.21, 95% CI: 1.26-8.85). Those neonates with low birth weight were 2 times more likely to have low Apgar score than neonates with normal birth weight (AOR = 1.65, 95% CI: 1.02-3.11) (see Table 5).

## 4. Discussion

The current study was conducted to assess the prevalence and factors associated with low fifth minute Apgar score among newborn delivered in Jimma University Medical Center. The proportion of low 5th minute Apgar score in this study was 11.5%. This result is in line with another institution-based study in North-West Ethiopia, Gondar [20]. But the current study was lower than a retrospective study done in Southwest Ethiopia, Jimma (35.7%). The difference could be due to variation in study design and sample size as well as time of study.

According to the current study, prolonged duration of labor is significantly associated with low fifth minute Apgar score. Similar finding is detected by Altman et al. in Sweden [21], by Salustiano et al. in Brazil [6], and by Gudayu in Ethiopia [20]. The reason for low Apgar score could be due to fetal distress resulted from prolonged duration of labor.

Low birth weight is significantly associated with low fifth minute Apgar score in present study; study done by Levy et al. [22] and by Gudayu in Ethiopia share similar finding [20].

Similarly, in current study, maternal khat chewing during pregnancy was significantly associated with low fifth minute Apgar score. Even though there is no study done so far that explained this relationship, the probable reason may be because khat chewing during pregnancy has negative impact on maternal and fetal well-being specifically resulting in neonate with low birth weight [23]; possible effect of maternal khat chewing on neonate was by disrupting normal neural tube development, undifferentiating brain vesicles, and causing incomplete closure of the brain flexures [24].

Postdelivery factors which may affect Apgar scoring and problems or defects in measuring were not considered and taken as limitation of this study. Because the factors affecting the Apgar score can vary from one health facility to other in the same country, multi-set-up study which may be at country level will be conducted in our future study.

## 5. Conclusion

In the current study, about one tenth of newborns had low 5th minute Apgar score. The likelihood of low fifth minute Apgar score were increased with prolonged duration of labor, history of mother's khat use, and low birth weight. Given the adverse impact of low fifth minute Apgar score on neonates, the authors of this study recommend abstinence from khat use and comprehensive interventions to reduce low birth weight.

## Figures and Tables

**Table 1:** Sociodemographic characteristics of newborn mothers delivered at Jimma University Medical Center, Southwest Ethiopia, 2018.

Variables	Frequency (%)
Maternal age	
≤20 years	38 (15.3)
21-34 years	263 (75.6)
≥35 years	47 (13.8)
Marital status	
Married	217 (62.4)
Widowed	19 (5.5)
Divorced	48 (13.8)
Single	64 (18.4)
Maternal education	
Illiterate	103 (29.6)
Primary education	101 (29)
Secondary education	94 (27)
Collage and above	50 (14.4)
Maternal occupation	
Housewife	135 (38.8)
Farmer	55 (15.8)
Government employee	88 (25.3)
Nongovernmental employee	27 (7.8)
Merchant	41 (11.8)
Others^∗^	2 (0.6)

^∗^Daily laborers.

**Table 2:** Maternal obstetric-related characteristics of newborn mothers delivered at Jimma University Medical Center, Southwest Ethiopia, 2018.

Variables	Frequency (%)
Mode of delivery	
SVD^¶^	149 (42.8)
Instrumental	98 (28.2)
Cesarean section	101 (29)
Condition of labor	
Spontaneous	249 (71.6)
Induced/augmented	99 (28.4)
Duration of labor	
Normal	295 (85)
Prolonged	53 (15)
History of spontaneous abortion	
Yes	22 (6.3)
No	326 (93.7)
History of stillbirth	
Yes	19 (5.5)
No	329 (94.5)
History of infant death	
Yes	27 (7.8)
No	321 (92.2)
Antenatal care follow-up	
Yes	317 (91.1)
No	31 (8.9)
Number of visits	
1 times	24 (6.9)
2-3 times	176 (50.6)
≥4 times	117 (33.6)
Complication during current pregnancy	
Yes	73 (21)
No	275 (79)
Type of complication	
Preeclampsia	26 (7.5)
Maternal infection	18 (5.2)
Antepartum hemorrhage	7 (2)
Others	22 (30.3)
Maternal chronic illness before conception	
Yes	91 (27)
No	257 (73)
Type of chronic illness	
Hypertension	33 (70.2)
Diabetes	20 (42.5)
Both	3 (6.3)

^¶^SVD: spontaneous vaginal delivery.

**Table 3:** General neonatal characteristics of newborn delivered at Jimma University Medical Center, Southwest Ethiopia, 2018.

Variables	Frequency (%)
Sex of neonate	
Male	170 (48.9)
Female	178 (51.1)
Gestational age at birth	
Term	225 (64.7)
Preterm	62 (17.8)
Postterm	61 (17.5)
Birth order	
First	76 (21.8)
Second	118 (33.9)
Third and above	154 (44.3)
Presentation at birth	
Vertex	226 (65)
Nonvertex	122 (35)
5^th^ minute APGAR score	
<7	40 (11.5)
≥7	308 (88.5)
Birth weight (kg)	
VLBW	1 (0.2)
LBW	21 (6)
NBW	283 (81.3)
HBW	43 (12.3)

**Table 4:** Behavioral and nutritional characteristics of newborn mothers delivered at Jimma University Medical Center, Southwest Ethiopia, 2018.

Variables	Frequency (%)
Cigarette smoking history	
Current	22 (6.3)
Former	15 (4.3)
Passive	47 (13.5)
Never	264 (75.9)
Khat chewing history	
Yes	56 (16.1)
No	292 (83.9)
Maternal folic acid use	
Yes	26 (7.5)
No	322 (92.5)
Maternal alcohol use history	
Yes	59 (17.0)
No	289 (83.0)

**Table 5:** Factors associated with low fifth minute Apgar score in newborn delivered at Jimma University Medical Center, Southwest Ethiopia, 2018.

Variables	Low 5^th^ minute APGAR score		Bivariate analysis		Multivariate analysis		
	Yes	No	Chi-square (*χ*^2^)	*P* value		AOR (95% CI)	*P* value
Maternal education							
Illiterate	12	91	1.84	0.113		0.32 (0.05-1.85)	0.205
Primary	13	88				0.33 (0.6-1.88)	0.216
Secondary	12	82				0.36 (0.05-2.26)	0.36
Collage and above	3	47		1		1	1
Gravidity							
Primigravida	13	67	2.31	0.129		1.66 (0.02-118.3)	0.815
Multigravida	27	241		1		1	
Duration of labor							
Normal	33	262	5.63	0.211		0.24 (0.22-0.55)^∗∗∗^	0.022^∗∗^
Prolonged	7	46		1		1	
History of stillbirth							
Yes	4	15	1.80	0.179		4.5 (0.09-2.20)	0.326
No	36	293		1		1	
Number of ANC visits							
1 times	7	17	12.34	0.113		2.71 (0.57-12.89)	0.210
2-3 times	19	157				2.36 (0.44-12.57)	0.314
>4 times	9	108				1	
Sex of neonate							
Male	13	157	4.83	0.028		1.48 (0.57-3.85)	0.417
Female	27	151		1		1	
Smoking							
Current	1	21	5.02	0.172		3.31 (0.43-25.41)	0.962
Former	1	14				2.21 (0.28-17.32)	0.347
Passive	2	45				3.55 (0.82-15.28)	0.269
Never	36	228		1		1	
Maternal khat use							
Yes	17	39	23.34	<0.00		3.21 (1.26-8.85)^∗∗∗^	0.024^∗∗^
No	23	269		1		1	
Presentation							
Vertex	23	203	14.68	0.014		0.85 (0.17-1.52)	0.216
Non-vertex	17	105		1		1	
Condition of labor							
Spontaneous	25	224	7.25	0.196		0.45 (0.22-3.41)	0.082
Induced	15	84		1			
Birth weight							
Low	18	4	35.97	0.011		1.65 (1.02-3.11)^∗∗∗^	0.048^∗∗^
Normal	22	304		1			

^∗∗∗^Statistically significant.

## Data Availability

The authors confirm that all data underlying the findings are fully available without restriction. All relevant data are within the manuscript.

## Abbreviations

ENND: Early neonatal death
ETB: Ethiopian birr
HBW: High birth weight
JUMC: Jimma University Medical Center
LBW: Low birth weight
MDG: Millennium development goal
NBW: Normal birth weight
SDGs: Sustainable development goals
SVD: Spontaneous vaginal delivery
VLBW: Very low birth weight.
